# The effect of education based on health belief model on promoting preventive behaviors of hypertensive disease in staff of the Iran University of Medical Sciences

**DOI:** 10.1186/s13690-021-00594-4

**Published:** 2021-05-05

**Authors:** Nemam Ali Azadi, Arash Ziapour, Javad Yoosefi Lebni, Seyed Fahim Irandoost, Jaffar Abbas, Fakhreddin Chaboksavar

**Affiliations:** 1 Department of Biostatistics, School of Health, Iran University of Medical Sciences, Tehran, Iran; 2 Research Center for Environmental Determinants of Health (RCEDH), Health Institute, Kermanshah University of Medical Sciences, Kermanshah, Iran; 3 Health Promotion Research Center, Iran University of Medical Sciences, Tehran, Iran; 4 Department of Public Health, School of Health, Urmia University of Medical Sciences, Urmia, Iran; 5 Antai College of Economics and Management/School of Media and Communication, Shanghai Jiao Tong University, Shanghai, China; 6 Nursing Care Research Center, Health Research Institute, Babol University of Medical Sciences, Babol, I.R. Iran

**Keywords:** Hypertension, Health Belief Model, Robust ANCOVA

## Abstract

**Background:**

Hypertension is one of the major causes of many diseases, such as heart attack, strokes, kidney failure, and many internal disorders. This presentresearch study aimed to investigate the impact of educational programs based on the health belief model to promote hypertension prevention behavior of Iran University of Medical Sciences staff.

**Methods:**

This study has incorporated pretest-posttest quasi-experimental based on 128 staff members and randomly assigned the recruited and involved participants to an intervention (*n* = 64) and a control group (n = 64). The data collection tool was based on a questionnaire related to health belief model constructs based on 42 questions. The study interpreted the results using ANCOVA and robust ANCOVA as suitable approaches.

**Results:**

ANCOVA showed improvement in the cues to participants’ action following educational interventional (*p* = 0.011). the robust ANCOVA specified that the intervention was successful for participants with low to moderate initial levels of knowledge, perceived susceptibility, perceived severity, perceived barriers, and self-efficacy scores. The levels of these components did not change in participants with very high baseline scores. Compared to a control group, regardless of baseline score, the perceived benefits and practice (behavior) of participants at the intervention group were improved significantly (*P* <  0.05).

**Conclusion:**

This current study specified that the education-based health belief model effectively promotes hypertension preventive behaviors among Iran University of Medical Sciences staff.

## Background

hypertension is the major cause of numerous diseases, including heart attacks, strokes, kidney failure, and many internal disorders [1]. The disease is common, asymptomatic, and may last for several years while the person is not aware of it [2, 3]. Hypertension has hit about 50 million Americans and more than 600 million people around the world. It is one of the most collective causes of adult visits to physicians [2]. Hypertension is the cause of 45% of myocardial infarction, 51% of stroke deaths, and 9.5 million deaths annually, and it is estimated to account for one-fourth of all deaths by 2030 [4]. In Irani adults, the prevalence of hypertension ranges between 25 to 35%, reportedly [5, 6]. The high prevalence of hypertension worldwide and its severe effects on the body’s organs have made this disease a significant health problem in all communities [7]. The prevalence of hypertension is still increasing in most parts of the world. It is estimated that this disease is growing in developing countries [8, 9], especially Asia and the Middle East, mainly due to lifestyle, especially high-calorie diets, and the use of ready-made and salty foods [10, 11]. According to Etaat et al., the study conducted in 2020, the leading cause of high blood pressure in Iran is obesity and high waist size [12]. After obesity, age, inactivity, high stress and occupational factors were the causes of hypertension, respectively [11]. Thus, past studies suggest promoting preventive behaviors for controlling blood pressure to prevent its complications through education. It needs to follow a healthy lifestyle and make changes to high-risk behaviors such as overeating, smoking, immobility, and adhering to mental health principles and avoiding stressful situations [13].

The value of educational programs depends on their effectiveness, which depends mainly on the correct use of theories and models in health education [14]. Selecting a proper educational model is the first step in the educational planning process [15]. The health belief model (HBM) refers to one of the affective educational models in preventing chronic diseases and health promotion and acted as a practical framework for designing educational interventions and promoting preventive behaviors [16, 17]. This model is a comprehensive model that plays a significant role in disease prevention. According to this model, a person’s decision and motivation to adopt a healthy behavior depends on three categories: personal perception, moderating behaviors, and the likelihood of doing that behavior [18]. This model includes the constructs of self-efficacy, perceived susceptibility, perceived severity, perceived barriers, perceived benefits, and cues to action [19]. The results of the studies indicate that the application of this model is successful so that in one study, the average score of the model constructs on blood pressure after the educational intervention showed a significant difference [19]. Due to the importance of education in promoting preventive behaviors against hypertension, this study was conducted to investigate the effect of educational programs based on health belief models on promoting behaviors to prevent hypertension in Iran University of Medical Sciences staff.

## Methods

### Study design

This study has incorporated pretest-posttest quasi-experimental based on 128 staff members and randomly assigned the recruited and involved participants to an intervention and a control group. The investigators conducted this study in 2019. The study comprised two randomly divided groups, interventional (*n* = 64) and controlled (n = 64). The investigators randomly allocated the samples between the two groups, the first sample was placed in the intervention group by lot, and then the samples were placed one by one in the groups. Sample size based on the mean and standard deviation of the structures of the health belief model from similar studies with standard deviation (s) = 2.31, reliability coefficient (z) = 1.96, accuracy (d) = 0.4, and using the formula to determine Cochran sample size $\boldsymbol{n}=\frac{{\boldsymbol{z}}^{\mathbf{2}}{\boldsymbol{s}}^{\mathbf{2}}}{{\boldsymbol{d}}^{\mathbf{2}}}$ One hundred twenty-eight people participated in the study. The data collection tool was a researcher-made questionnaire based on HBM constructs, which included 42 questions. The questionnaire included questions about demographic characteristics (5 questions), knowledge (5 questions, Minimum score 0, and maximum score 5). Besides, HBM had perceived susceptibility (4 questions, Minimum score four, and top score 20). For instance, one of the questions was: I may suffer from the Complications of high blood pressure in the future—perceived severity (4 questions, Minimum score four, and maximum score 20). One of the questions was: having the Complications of high blood pressure can cause a heart attack—perceived barriers (6 questions, Minimum score six, and maximum score 30). For example, one of the questions was: the cost of going to the doctor is high for me. Perceived benefits (5 questions, Minimum score five, and maximum score 25): For example, preventing high blood pressure reduces anxiety and stress in me. Self-efficacy (4 questions, Minimum score four, and the maximum score 20), For example, one of the questions was: I can avoid eating salt despite my interest in it. Cues to action (3 questions, Minimum score 0 and maximum score 3): do you seek information from sources such as doctors and other health care staff to prevent high blood pressure? And Practice (6 questions, Minimum score six and maximum score 18) For example, one of the questions was: do you exercise to prevent high blood pressure? Susceptibility, severity, benefits, and barriers constructs were 5-points Likert scale, ranging from strongly agree, agree, no comment, disagree, and strongly disagree. The practice questions were 3-points Likert scale, ranging from always, rarely, and sometimes. The cues to action questions were binary questions, yes or no, and participants’ knowledge was assessed using true or false questions.

The educational intervention is planned as an initial phase followed by five training sessions (60 min per session) covering fat consumption, salt consumption, weight control, exercise, and stress management. The investigators designed training sessions using group discussion, lecture, question and answer techniques. At the baseline, end of the intervention, and three months after the educational intervention, participants were asked to complete the questionnaire.

### Statistical analysis

The study results indicated a decent scores of mean ± SD and specified adequate outcomes. Mann-Whitney test was used to compare the levels of the quantitative variable between two groups. The study measured a chi-squared test for categorical variables of the selected model. Analysis of covariance (ANCOVA) was used to test for differences in component means among the groups by adjusting the effect of components at baseline. ANCOVA results are reliable if i) the relationship between the component levels at the end of the study and baseline does not differs across the groups (known as the homogeneity of regression slopes), and ii) the independence of the baseline scores and study groups is met. The first assumption was investigated using the Mann-Whiney test. The second assumption was verified by augmenting an interaction term (baseline × group effect) to the model. This assumption is violated if the interaction effect was significant. In terms of violation of ANCOVA assumptions, robust ANCOVA was used. All statistical analysis was performed using R statistical software and the WRS2 package [20].

## Results

Of 128 participants, 55 (43%) were men, and 73 (57%) were women. The mean age of participants was 40.98 ± 8.75 (24 to 58 years). Moreover, 15.6% of participants had a high school degree, and the remaining, 84.4%, had an academic degree. Eighteen subjects (14%) reported blood pressure history. Demographic characteristics of participants in terms of study groups are given in Table 1. The comparison of participants’ characteristics revealed no statistically significant difference in the age (Mann-Whitney test, *P* = 0.241), work experience (Mann-Whitney test, *P* = 0.363), gender (Chi-squared test, *P* = 0.858), blood pressure history (Chi-squared test, *P* = 0.611), and their education levels (Chi-squared test, *P* = 0.572) between the cases and controls.

**Table 1:** Demographic characteristics of case and control groups. Study on the effect of education on the hypertension preventive behaviors among the staff of the Iran University of Medical Sciences, during 2019

Characteristic	Cases	Controls	*P*-value
Age (years)			
Mean ± SD	41.94 ± 9.04	40 ± 8.38	0.241^a^
Work experience (years)			
Mean ± SD	15.98 ± 8.99	14.41 ± 8.80	0.363^a^
Gender			
Male	28 (43.8%)	27 (56.3%)	0.858^b^
Female	36 (42.2%)	37 (57.8%)	
BP history			
Yes	10 (15.6%)	8 (12.5%)	0.611^b^
No	54 (84.4%)	56 (87.5%)	
Education			
High School diploma	12 (18.8%)	8 (12.5%)	0.572^b^
Undergraduate degree	29 (45.3%)	29 (45.3%)	
Postgraduate degree	23 (35.9%)	27 (42.2%)	

^a^ Mann-Whitney test was used,

^b^ Chi-squared test was used

Since we used a researcher-made questionnaire to measure the knowledge, perceived susceptibility, perceived severity, self-efficacy, perceived benefits, perceived barriers, and cues to the action of subjects, the reliability of the questionnaire has to be reported. Cronbach’s alpha, the most common measure of internal consistency, was used to obtain the reliability of the questionnaire in measuring the components mentioned above (Table 2). Cronbach’s alpha was calculated twice; i) using the baseline data (*n* = 128) and ii) using the data available at the end of the study. It turns out that reliability was acceptable at most dimensions except for perceived benefits/barriers measured at baseline, where Cronbach’s alpha was well below 70% threshold.

**Table 2:** Cronbach’s alpha for measuring internal consistency of the dimensions of the questionnaire using the participants’ information at the baseline and the end of study among the staff of the Iran University of Medical Sciences in 2019

Construct (Component)	Cronbach’s α	
	Baseline (*n* = 128)	End of study (*n* = 128)
Knowledge	0.92	0.72
Perceived Susceptibility	0.58	0.61
Perceived severity	0.72	0.68
Perceived barriers	0.53	0.60
Perceived benefits	0.50	0.61
Cues to action	0.84	0.78
Self-efficacy	0.70	0.60
Practice	0.70	0.80

The study shows the mean and standard deviation of the questionnaire (components) for each group related to the baseline, as shown in Table 3. Since the normality assumption was violated, the Mann-Whitney test was used to compare the differences between groups. The results revealed no statistically significant difference in component levels between the two groups at baseline. At the end of the study, the differences between case and control groups were all statistically significant except for the ‘Cues to action’ component (Mann-Whitney test, *P* = 0.914).

**Table 3:** Comparing components between cases and controls group at baseline and the end of study among the Iran University of Medical Sciences staff in 2019 using Mann-Whitney test. No difference between participants at baseline, but significant differences were observed after intervention at most components

	Baseline			End of study		
Component	Case	Control	*P*-value	Case	Control	*P*-value
Knowledge	2.45 ± 2.14	2.59 ± 2.12	0.503	3.44 ± 1.76	2.55 ± 2.22	0.011
Perceived susceptibility	11.88 ± 2.42	11.52 ± 2.38	0.451	13.27 ± 1.65	11.56 ± 2.30	< 0.001
Perceived severity	13.42 ± 2.58	13.53 ± 2.61	0.780	15.0 ± 1.51	13.66 ± 2.41	0.001
Perceived barriers	19.75 ± 3.01	19.53 ± 3.16	0.625	16.85 ± 2.12	19.62 ± 3.15	< 0.001
Perceived benefits	16.22 ± 2.23	16.58 ± 2.37	0.316	18.16 ± 1.34	16.33 ± 2.31	< 0.001
Cues to action	1.94 ± 1.22	2.0 ± 1.26	0.596	2.03 ± 1.13	1.95 ± 1.24	0.914
Self-efficacy	13.12 ± 2.24	13.55 ± 2.18	0.251	14.61 ± 1.12	13.64 ± 1.92	0.005
Practice	9.25 ± 1.91	9.33 ± 1.84	0.817	12.59 ± 0.79	9.25 ± 1.78	< 0.001

Although the Mann-Whiney test confirmed the effectiveness of the educational intervention, it can be misleading as it fails to control for the level of components at the baseline. Thus, ANCOVA was used to test for differences in component means among the groups by adjusting the effect of components at baseline. The assumption of homogeneity of regression slopes and the independence of baseline scores and study groups were tested using Mann-Whitney and interaction effects. Results showed that these assumptions only met for the ‘cues to action’ component, and for other features, the latter assumption was violated. Thus, ordinary ANCOVA was used to interpret the results of the ‘Cues to action’ component, but for other components, robust ANCOVA [21] was used. The results of performing ordinary ANCOVA showed that educational intervention increased the ‘Cues to action’ in the case group (F (1,124) =0.59, *P* = 0.011) compared to the control group [22].

In Robust ANCOVA, the trimmed means (20%) were compared between two groups at some design points (usually five points), where the relationship between pre and post values was the same in both groups. Comparisons between trimmed means of case and control groups, ${\overline{x}}_{case}^t-{\overline{x}}_{ctrl}^t$ The investigators set it by constructing 95% confidence intervals using the bootstrapping method. Confidence intervals were adjusted for inflation type I error in multiple comparisons. Table 4 represents the results of performing robust ANCOVA for knowledge, perceived susceptibility, and perceived severity components. In this Table, at each design point, *n*_1_ and *n*_2_ denote the sample sizes used to obtain trimmed means at the case and control group, respectively. The significant results are displayed in the bold face under the 95% CI column (Table 4). It appears that the educational intervention raised the knowledge score of those participants who had lower initial scores (0 or 3) at the baseline. Still, it didn’t affect the knowledge of participants with high scores as large as 5. Furthermore, intervention affected participants with initially perceived susceptibility scores between 7 to 11 and a perceived severity score less than 13 at the baseline. In other words, the intervention was quite successful for participants who had low scores at the beginning of the study.

**Table 4:** The result of performing robust ANCOVA for knowledge, perceived susceptibility, and perceived severity among the Iran University of Medical Sciences staff during 2019. Significant results are highlighted in boldface intervals

Variable	Design point	n1	n2	${\overline{x}}_{case}^p-{\overline{x}}_{ctrl}^p$	95% CI
Knowledge	0	30	30	1.67	(0.87, 2.46)
	3	47	18	1.19	(0.20, 2.59)
	5	33	33	0.38	(0.01, 0.77)
Perceived susceptibility	7	13	13	3.33	(4.17, 2.50)
	10	40	38	2.04	(3.01, 1.07)
	11	37	42	1.65	(2.59, 0.71)
	14	31	35	0.42	(1.38, 0.53)
	16	18	18	0.92	(1.86, 0.03)
Perceived severity	9	19	17	2.66	(3.59, 1.72)
	11	25	28	2.69	(3.76, 1.62)
	14	39	40	0.79	(1.62, 0.06)
	16	37	33	0.64	(1.34, 0.06)
	18	18	15	0.17	(0.97, 0.64)

**Fig. 1:**
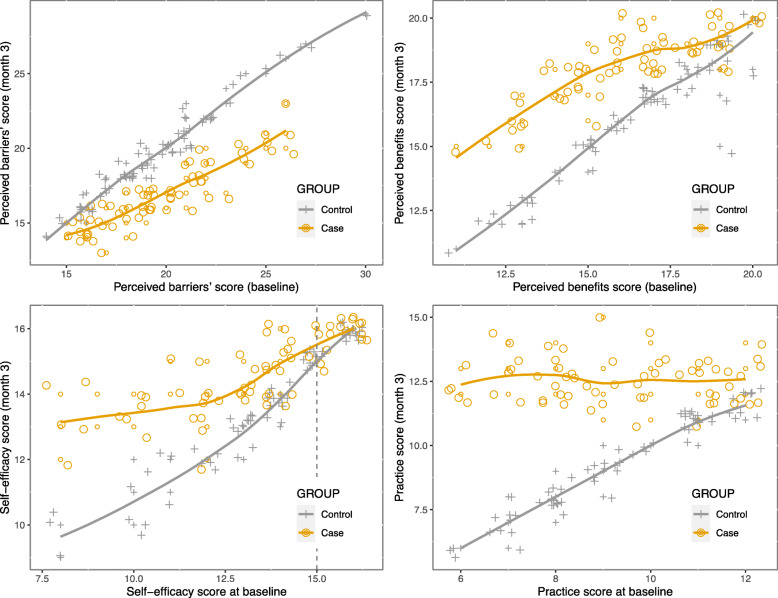
The plot of perceived barriers, perceived benefits, self-efficacy, and practice scores at baseline (on the x-axis) against post-perceived barriers, post-perceived benefits, post-self-efficacy, and post-practice scores (on the y-axis) from robust ANCOVA. Two regression lines represent the intervention group (orange line, circle points) and the control group (dark line, plus symbols)

The investigators emphasized avoiding a lengthy Table for the remaining components (perceived barriers, perceived benefits, self-efficacy, and Practice) and displayed the results of robust ANCOVA and plotted in Fig. 1. In this Figure, the vertical dashed lines represent a point where the differences between two groups from this point onward become non-significant. For instance, at panel A (Fig. 1), which displays the scatter plot of the perceived barriers scores at baseline and the end of the study, the educational intervention decreased significantly the perceived barrier scores of all participants. Still, in terms of self-efficacy, it was successful for those participants recognized with an initial score less than 15 at baseline (non-significant results appeared only from 15 onward, which compromises 28% of participants) (Fig. 1, panel C). In other words, an intervention was successful in increasing the self-efficacy score for 72% of subjects.

The educational intervention was also quite effective in promoting the ‘Perceived benefits’ (panel B, Fig. 1) and ‘Practice’ score (panel D, Fig. 1) of individuals (scores were significantly higher at case group at all design points).

## Discussion

The present study results revealed that the educational intervention based on HBM successfully increased perceived susceptibility, perceived severity, perceived barriers, perceived benefits, self-efficacy, cues to action, and practice scores of participants. The findings agreed with Sadeghi et al. study [23] and Ardabili et al. study [24]. Abood et al. also surveyed to apply the health belief model on University staff and showed that educational intervention significantly increased knowledge in the intervention group [25]. The present study results showed that the mean scores of perceived susceptibility and perceived severity increased substantially after an educational intervention. These results were in line with the Sharifi Darani et al. study [26]. In the survey conducted by Baghiani et al., the educational intervention significantly increased the susceptibility and the perceived severity scores in the experimental group [27].

Also, in terms of the perceived severity, our results were consistent with Cherkzy et al. [28] and Azadbakht et al. [29] studies. Still, they were inconsistent with Mohammadi et al. [30] study who reported lower perceived severity scores after intervention. In justifying this issue, it can be stated that education promotes the perceived susceptibility and subsequently increases the perceived severity in the intervention group [31]. University staff after education found the belief that if non-compliance with preventive behaviors and lack of Blood pressure control is at risk for high blood pressure. They understand the depth of the risk and the seriousness of the complications in physical, psychological, social, and economic terms. The present study results showed that the mean score of perceived benefits in the intervention group increased significantly after intervention. This result was consistent with that of the study conducted by Amodeo et al. [30]. Likewise, the present study results were consistent with those of the survey conducted by Zeinaly et al. [32].

The present study results showed that the mean score of the perceived barriers after the educational intervention was significantly different in the intervention group. Still, it did not show a significant difference in the control group. This result was consistent with that of the study conducted by Mohammadi et al. [30]. Tan showed that perceived barriers led to reduced adherence to medical orders, such as regular use of medications to control hypertension [33]. Chao et al. also reported an inverse relationship between perceived barriers and health behaviors, meaning that increasing the perceived barriers increases the probability of health behaviors [34]. In the present study, the mean self-efficacy score increased after educational intervention in the intervention group. The results of a review study conducted by Yehle and Plake showed that both short-term and long-term educational interventions could improve patients’ self-efficacy [35]. Similar studies also revealed the effect of education on the health belief model in enhancing the mean score of self-efficacy [36, 37].

The results of the Mann-Whitney test revealed no significant difference between two groups of intervention and control in terms of Cues to action dimension score before and after education. This result was consistent with that of the study conducted by Mohammadi et al. [38]. Still, it was inconsistent with the research undertaken by Amini et al. [38] and Sadeghi et al. [38]. Finally, the mean score of Practice (behavior) increased after the educational intervention in the intervention group. The results of the studies conducted by Amodeo et al. [38] confirm the present study results.

## Conclusion

This study shows that education based on the health belief model effectively promotes hypertension preventive behaviors in University staff. Therefore, by enhancing the knowledge level, perceived susceptibility, perceived severity, perceived benefits, and staff self-efficacy, it is possible to affect their behavior positively.

## Data Availability

The datasets using in the study are available from the corresponding author on reasonable request.

## Abbreviations

EBHBM: Education-based health belief’s model
PC: Preventive behaviors
HD: Hypertension disease
